# Serotonin transporter deficiency alters socioemotional ultrasonic communication in rats

**DOI:** 10.1038/s41598-019-56629-y

**Published:** 2019-12-30

**Authors:** Joanna Golebiowska, Małgorzata Hołuj, Agnieszka Potasiewicz, Diana Piotrowska, Agata Kuziak, Piotr Popik, Judith R. Homberg, Agnieszka Nikiforuk

**Affiliations:** 1 Maj Institute of Pharmacology Polish Academy of Sciences, Department of Behavioral Neuroscience and Drug Development, Kraków, Poland; 2 Department of Cognitive Neuroscience, Donders Institute for Brain, Cognition and Behaviour, Radboud University Medical Center, Nijmegen, The Netherlands

**Keywords:** Behavioural methods, Social behaviour

## Abstract

It has been widely established that serotonin plays important role in the regulation of emotional and social behaviour. Rodents with a genetic deletion of the serotonin reuptake transporter (SERT) are used as a model to study lifelong consequences of increased extracellular 5‐HT levels due to its impaired reuptake. SERT knock-out (SERT-KO) mice and rats consistently showed anxiety-like symptoms and social deficits. Nevertheless, the impact of SERT deletion on socioemotional ultrasonic communication has not been addressed. Here we investigated the impact of lifelong serotonin abundance on ultrasonic vocalisation accompanying social interactions and open field exploration in rats. SERT-KO rats displayed reduced overall duration of social contacts, but increased time spent on following the conspecific. The altered pattern of social behaviour in SERT-KO rats was accompanied by the structural changes in ultrasonic vocalisations, as they differed from their controls in distribution of call categories. Moreover, SERT deletion resulted in anxiety-like behaviours assessed in the open field test. Their anxious phenotype resulted in a lower tendency to emit appetitive 50-kHz calls during novelty exploration. The present study demonstrates that genetic deletion of SERT not only leads to the deficits in social interaction and increased anxiety but also affects ultrasonic communication.

## Introduction

The ability to communicate is crucial to establish and maintain social functioning in everyday life. The persistent deficits of social communication are now a growing health and social concern throughout the world. Studying social communication in preclinical settings is also possible, since rodents vocalise in the ultrasonic range^1^. This phenomenon is increasingly used as a readout for communication impairments in rodent models of neurodevelopmental disorders^2,3^. The call rate and the frequencies of emitted ultrasonic vocalisations (USVs) depend on the age and emotional state and are modulated by social context. In adult laboratory rats, two main types of USVs have been described: the relatively low (22-kHz) and high (50-kHz) frequency calls^4^. The 22-kHz call type, termed as “alarm” vocalisations, have been associated with emotionally negative social experiences such as encounter with a predator or an aggressive conspecific^5^. The 50-kHz “happy calls” may be detected in appetitive settings, including amicable social interactions^6^.

Digital sound spectrographic analysis provides more detailed information about USVs structure and thereby allows identifying multiple call categories within the rich repertoire of rat 50-kHz calls^4,7^. Based on their characteristics, the calls can be separated into the “flat” calls (with a near-constant frequency), and frequency-modulated (FM) calls. The most characteristic FM calls are the “trills” that appear in spectrograms as rhythmic waves of ups and downs. Other FM calls include “one-component” calls (characterised by variable changes with ascending/descending constant pattern, typically categorised as “complex”, “ramp” or “inverted U-shape” calls) and multicomponent calls that comprise two or more sounds (typically categorised as “step”, “multistep” or “composite” calls). While the precise meaning of these USV call categories remains to be established, the detailed characteristics of sonographic patterns may provide more comprehensive assessment of rodents’ socioemotional state than by using purely quantitative measures.

One molecule that plays an important role in the regulation of emotional and social behaviour is serotonin (5-hydroxytryptamine; 5-HT). The 5-HT system is implicated in various neuropsychiatric conditions including mood, anxiety and autism spectrum disorders^8–10^. A key regulator of serotonin neurotransmission is the serotonin reuptake transporter (SERT) which transports 5-HT from the synaptic cleft back into the pre-synaptic terminal^11^. SERT is transiently expressed in many brain regions during embryonic developmental periods^12^. Several lines of evidence indicate that early life pharmacological SERT inhibition can impair socioemotional behaviour^12^ due serotonin’s neurotrophic actions in brain development and consequent structural changes^8,12–14^. Accordingly, maternal selective serotonin reuptake inhibitor (SSRI) treatment has been linked to the changes in social behaviour in both preclinical and clinical studies^15–17^. This should not be confused with acute SSRI treatment in adults, which bypasses the developmental period and induces different and sometimes even opposite behavioural changes compared to early life SSRI exposure^18^. SERT functioning is affected by genetic factors, such as the SERT polymorphism in humans. One of the most widely studied polymorphism occurs within the promoter region of the SERT gene (SLC6A4)^19–21^. The resulting short allelic variant is associated with decreased expression and function of SERT and affects emotional regulation, anxiety-related and social behaviour^22^. This human polymorphism can be mimicked by the genetic deletion of the SERT in rodents, which are used as a model to study lifelong consequences of increased extracellular 5‐HT levels due to its impaired reuptake^23^. Given that early pharmacological SERT inhibition and genetic SERT knockout have overall similar effects on socioemotional behaviour, it is thought that the behavioural changes observed in genetic animal models lacking SERT are to a substantial part due to changes in brain development^13^.

Studies conducted in SERT knock-out (SERT-KO) mice and rats consistently showed anxiety-like symptoms^24^ and social deficits^25^. Nevertheless, the impact of SERT deletion on socioemotional ultrasonic communication has not been widely addressed. Since rats exhibit more complex social behaviour and a richer acoustic communication system compared to mice^26^, we took advantage of SERT deficient rats to investigate quantitative and structural changes of USVs emitted during reciprocal social interactions. We also correlated the rats’ USVs emitted during the open field exposure with their anxious phenotype. To complement the characteristics of social deficits in SERT-KO rats, we assessed the sociability and preference for social novelty in the three-chamber test that allows for minimizing the active influence of the social partner. Finally, their anxious phenotype was confirmed in the elevated plus maze test.

## Results

### Exploratory behaviour in the open field

**Figure 1:**
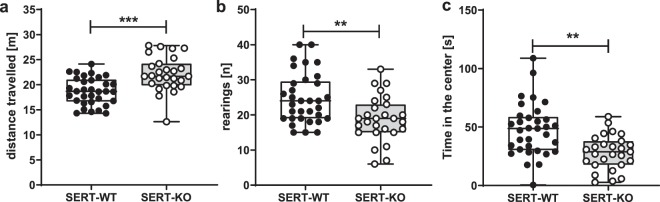
SERT-KO rats demonstrated altered exploratory behaviour in the open field test. The total distance travelled (**a**), number of rearings (**b**) and time spent in the center of the open field (**c**). Data are presented as median (horizontal line), interquartile range (box) and minimum and maximum values (whiskers). N = 33 (SERT-WT) and N = 27 (SERT-KO) rats per group. Symbols: **p < 0.01, ***p < 0.001; intergenotype comparison, Student’s t-test (a,c) or Mann-Whitney U Test (b).

The SERT-KO rats demonstrated longer total distance travelled (t = 4.361, df = 58, **p** < **0.001**, Student’s t-test; Fig. 1a), reduced number of rearings, (Z = 2.807, **p** = **0.009**, Mann-Whitney U Test; Fig. 1b) and less time spent in the center of the open field (t = 3.863, df = 58, **p** = **0.001**, Student’s t-test; Fig. 1c) compared to wild-type controls.

### Novelty exploration-induced ultrasonic vocalisations

The analysis of USV emission during the open field test revealed that the relation of 22-kHz emitting to non-emitting rats did not significantly differ between groups (SERT-WT: 11/22 and SERT-KO: 4/23, Chi^2^ = 2.716, **NS**; data not shown). However, significantly less SERT-KO animals emitted 50-kHz calls as compared to SERT-WT group (SERT-WT: 24/9 and SERT-KO: 12/15, Chi^2^ = 4.949, **p** = **0.039**; Supplementary Fig. 1 S1). The average number of 50-kHz calls in USV-emitting animals did not differ between groups (U = 19, NS, Mann-Whitney U Test; Supplementary Fig. 2 S1), but calls emitted by SERT-KO rats were characterised by longer duration and a wider bandwidth (Supplementary Table 1 S1). The number of 50-kHz calls in USVs-emitting rats was positively correlated with time spent in the center of the open field in SERT-WT rats (r = 0.433, p = 0.034; Spearman rank correlation test), but not in SERT-KO rats (r = −0.098, NS).

### Social interactions

**Figure 2:**
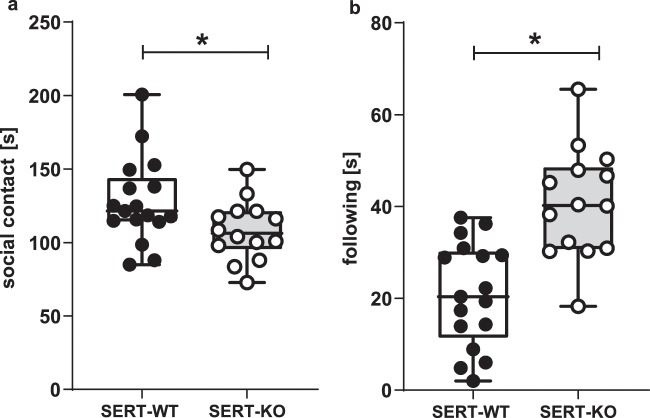
In the social interaction test, SERT-KO rats spent less time on social contact (**a**), but more time on following a social partner (**b)** as compared to SERT-WT rats. The total time spent on social contacts (**a**) and on following behaviour (**b**). Data are presented as median (horizontal line), interquartile range (box) and minimum and maximum values (whiskers). N = 17 (SERT-WT) and N = 14 (SERT-KO) pairs per group. Symbols: *p < 0.05 (Newman-Keul’s post-hoc test).

SERT-KO rats spent less time on social contact with the test partner as compared to SERT-WT controls (**p** = **0.014**, Newman-Keul’s post-hoc test following a significant genotype x behaviour type interaction: F[1,29] = 17.78, p < 0.001; Fig. 2a). However, the deletion of SERT increased the duration of the following behaviour (**p** = **0.014**, Newman-Keul’s post-hoc test; Fig. 2b).

### Social interaction-induced ultrasonic vocalisations

All pairs of rats emitted 50-kHz calls, and the relation of 22-kHz emitting to non-emitting rats did not differ between groups (SERT-WT: 9/8 and SERT-KO: 6/8, Chi^2^ = 0.31, NS). As demonstrated in Supplementary Fig. 1S2, SERT-KO rats did not differ from their wild type controls in terms of the number of emitted 50-kHz calls (t = 1.424, df = 29, **NS**) and 22-kHz calls (U = 19, **NS**). There was also no effect of genotype on proportion of alarm and appetitive calls (SERT-WT: 95.3% (50-kHz) and 4.7% (22-kHz) vs SERT-KO: 96.1% (50-kHz) and 3.9% (22-kHz), respectively).

**Figure 3:**
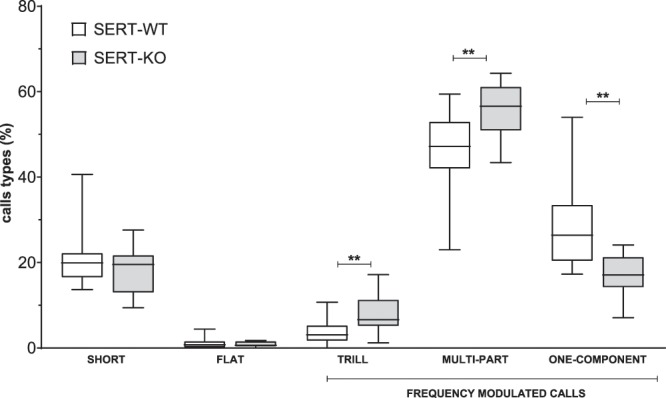
SERT-KO demonstrated changes in percentage distribution of FM 50-kHz call categories. Data are presented as median (horizontal line), interquartile range (box) and minimum and maximum values (whiskers). N = 17 (SERT-WT) and N = 14 (SERT-KO) pairs per group. Symbols: **p < 0.01 (intergenotype comparison, Student’s t-test).

However, the deletion of SERT affected the distribution of the 50-kHz USV categories (Fig. 3). Specifically, SERT-KO rats demonstrated an increased percentage of trill calls (t = 3.274, df = 29, **p** = **0.006**) and of multi-part calls (t = 3.436, df = 29, **p** = **0.005**) and a decreased percentage of one-component calls (t = 4.079, df = 29, **p** = **0.001**) as compared to WT controls.

The acoustic parameters (duration, bandwidth, and peak frequency) of 50-kHz call categories did not differ between genotypes (Supplementary Table 1 S2). Due to the lack of emission of a specific USV category, several pairs were excluded from the analysis of the peak frequency, bandwidth and call duration of a given call subtype (detailed description in Supplement 2). However, those pairs were included in the analysis of call type distribution (Fig. 3).

To test whether there is a relationship between the duration of social behaviour and the emission of 50-kHz calls, correlation analyses were performed (see Supplementary Table 2 S2). There was a positive correlation between the duration of the following behaviour and the total number of emitted 50-kHz calls in SERT-KO rats (r = 0.552, p = 0.041) but not in SERT-WT rats (r = 0.152, NS). The analyses of specific call categories revealed positive correlation between the number of short calls and the following behaviour (r = 0.723, p = 0.03) as well as between multi-part calls and the following behaviour (r = 0.547, p = 0.043) in SERT-KO rats. In contrast, the duration of social contact behaviour was negatively correlated with the number of flat calls (r = −0686, p = 0.002) in SERT-WT rats. No other significant correlations were found in either SERT-WT or SERT-KO rats.

### Sociability and social novelty preference

**Figure 4:**
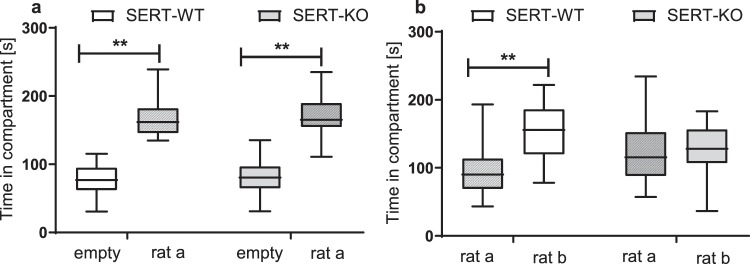
SERT-KO rats displayed intact sociability (**a**), but impaired social novelty preference (**b**). The total number of the time spent in the compartment with a stranger rat vs. an empty chamber (**a**), and in the compartment with a novel stranger vs. the first unfamiliar rat (**b**). Data are presented as median (horizontal line), interquartile range (box) and minimum and maximum values (whiskers). Part a (sociability): N = 33 (SERT-WT) and N = 27 (SERT-KO) rats per group. Part b (novelty preference): N = 30 (SERT-WT) and N = 23 (SERT-KO) rats per group. Symbols: **p < 0.01 (Newman-Keul’s post-hoc test).

SERT-WT and SERT-KO rats did not differ in their sociability, as demonstrated by a significant preference for the compartment containing the stimulus (a) rat vs. the empty chamber (**p** = **0.001**, Newman-Keul’s post-hoc test following a significant effect of the compartment: F[1,58] = 195.8, p < 0.001; Fig. 4a). In the second part of the test, SERT-WT demonstrated a preference for the compartment containing the novel rat (b) vs. previously encountered (a) rat (**p** = **0.004**, Newman-Keul’s post-hoc test following a significant compartment x genotype interaction: F[1,51] = 5.03, p = 0.029; Fig. 4b). The novelty preference was not observed in SERT-KO rats.

There were no significant differences between SERT-WT and SERT-KO rats in the number of entries into the chambers during the sociability and the social novelty preference (Supplementary Fig. 1 S3 and Fig. 2 S3). There were also no significant correlations between the total number of entries and the distance travelled that was independently measured during open field test (Supplementary Table 1 S3).

Due to the lack of exploration in the novelty preference test, 3 SERT-WT and 4 SERT-KO rats were excluded from the analysis.

### Elevated plus maze test

**Figure 5:**
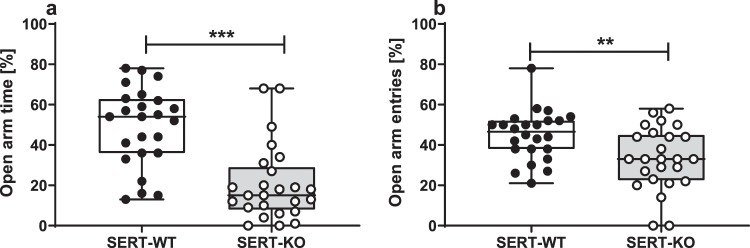
SERT-KO rats exhibited anxious-like behaviour in the elevated plus maze test. The percentage time spent in the open arms (**a**) and open arm entries (**b**). Data are presented as median (horizontal line), interquartile range (box) and minimum and maximum values (whiskers). N = 24 (SERT-WT) and N = 25 (SERT-KO) rats per group. Symbols: **p < 0.01, ***p < 0.001 (intergenotype comparison, Mann-Whitney U Test (**a**) and Student’s t-test (**b**)).

SERT-KO rats spent less time on the open arms (Z = 4.221, **p < 0.001**, Mann-Whitney U Test; Fig. 5a) and exhibited less open arm entries (t = 2.879, df = 47, **p = 0.006**, Student’s t-test; Fig. 5b) compared to the wild-type controls.

## Discussion

Present results demonstrate that an altered pattern of social interactions in rats lacking SERT is accompanied by the structural changes in ultrasonic vocalisations. Specifically, SERT-KO rats differed from their wild type controls in percent distribution of call categories within the frequency modulated 50-kHz class. Moreover, the “anxious” phenotype of SERT-KO observed in the open field was associated with a lower tendency to emit appetitive 50-kHz calls. Social deficits and anxious-like behaviour in SERT-KO rats were also observed in the social novelty preference and elevated plus maze tests, respectively.

Altered locomotor patterns and aberrant organisation of exploratory behaviour have been previously demonstrated in SERT-KO rats and mice^27–29^. Based on these data, it may be hypothesized that increased distance travelled by the SERT-KO rats in the present open field study is related to the altered exploratory strategies that involve meandering, increased angular velocity and intermittent exploratory activity^27^. In line with previous data, SERT-KO rats also demonstrated reduced vertical exploration, as indicated be reduced number of rearing episodes. Moreover, the reduced time spent in the center of the open field agrees with the well-documented anxious phenotype of SERT-KO animals^27–29^. The increased anxiety was also confirmed in another test based on the aversion to open areas, that is, in the elevated plus maze test.

As previously demonstrated^30^, the deletion of SERT reduced social contacts. However, SERT-KO rats spent more time following a social partner. Similar increases in following behaviours were previously noted in adolescent SERT-KO rats despite the deficient social play^31^. It does not appear that the observed effects are exclusively related to the increased locomotor activity of SERT-KO rats, because a positive correlation was found between the duration of the following behaviour and the emission of affective 50-kHz calls. Moreover, despite reduced reciprocal social interactions, the deletion of SERT did not affect sociability in a three-chamber test. These results may seem contradictory, but given that social interactions were assessed in the pairs of the same genotype, it is likely that the phenotype of the partner influenced social behaviour. To the contrary, the active influence of the social object is minimized in a three-chamber test. In line with our data, intact social approach was also demonstrated in SERT-KO mice^32^ (but see^33,34^ who reported opposite results). Thus, it may be concluded that the altered pattern of social interactions in SERT-KO rats do not necessarily indicate a general impairment of social affiliation. In line with this assumption, increased socio-positive behaviours were noted when studying long-term home-cage behavioural structure in SERT-KO mice^35^ and reduced aggression was demonstrated in both SERT-KO mice and rats in resident-intruder protocols^36,37^. It cannot be excluded that a reduction in social interactions results from enhanced levels of anxiety.

Nevertheless, the deletion of SERT may affect social cue processing, as indicated by reduced social novelty preference in SERT-KO rats. This deficit does not appear to result from altered exploratory activity, as the number of entries did not differ between the two compartments and did not correlate with the rat’s locomotor activity independently measured in the open field. Although it cannot be excluded that this deficit arises from a more general cognitive dysfunction, the novel object recognition was disrupted in SERT-KO rats only when tested with a long delay (i.e., 8 h)^38^. Alternatively, increased neophobia found in SERT-KO rats^28^ may account for the reduced novelty preference.

Although ample evidence indicates that there is a link between SERT abnormalities and emotional/social deficits, little is known about the role of SERT in the production of “aversive” 22-kHz and “happy” 50-kHz USVs. Recently, the increases in isolation-induced distress calls has been demonstrated in heterozygous SERT-KO rat pups^39^. Similar effects that may be interpreted as an increase in anxiety were previously produced by prenatal SSRI treatment^40^. However, low emission of 22-kHz calls was observed in adult rats in the current study. There were also no differences between SERT-WT and SERT-KO rats. It may not be surprising, since the used experimental set-up did not involve aversive stimuli and therefore did not allow for revealing any potential changes in 22-kHz USVs. We also did not observe less aggressive/more submissive behaviours that have been previously demonstrated for SERT-KO rats in the resident-intruder paradigm^37^ and could affect emission of alarm calls^41^.

To our knowledge, the emission of 50-kHz calls has not been evaluated in SERT-KO rats or mice. Little is also known about the role of serotonin in their production (reviewed in^42^). No effect was observed after administration of 3,4 methylenedioxymethamphetamine (MDMA), the compound that acts mainly by promoting the release of 5-HT from synaptic terminals^43^. Moreover, USVs were unchanged during social contacts of mice with a brain-specific serotonin depletion caused by a tryptophan hydroxylase 2 (TPH2) deficiency^44^. However, experimental data suggest that at least some of the 5-HT receptor subtypes may play modulatory role in the USVs emission. Accordingly, the 5-HT2C receptor antagonist evoked 50-kHz USVs and led to further increase of amphetamine-induced affective calls^45^. The opposite effect was noted for the agonist of 5-HT2C receptors. Interestingly, manipulation of 5-HT2C receptors predominantly affected frequency modulated calls, mostly of the trill category^45^. Moreover, administration of a full agonist of 5-HT1A receptors affected USVs emission in an inverted U-shape manner, suggesting that an optimal balance between activation of presynaptic and postsynaptic 5-HT1A receptors is necessary for call production^46^. This finding may be of particular interest given that the downregulation of expression and functions of the 5-HT2C and 5-HT1A receptors has been demonstrated in SERT-KO rats and mice^47,48^. Nevertheless, we did not observe significant changes between SERT-KO and SERT-WT rats in the number of emitted calls during social interactions. It cannot be excluded that the introduction of the isolation period before testing, that is known to increase social motivation, could further increase 50-kHz and thereby reveal potential inter-genotype differences. Despite the lack of quantitative differences in call emission, the deletion of SERT affected the distribution of the USV categories within the frequency modulated 50-kHz calls. Interestingly, increases were noted in the proportion of one of the most characterised frequency modulated USVs categories, that is the trill calls. However, while there is still a debate on the behavioural significance of trill calls, these calls have been most consistently associated with positive emotional state occurring during social situations and reflect rewarding properties of amphetamine^7^. Thus, it may be assumed that SERT deletion is associated with increased rewarding value of social contacts. This explanation agrees well with the increased sensitivity to psychosocial factors in both s-allele carriers and SERT knockout rodents^22^. Alternatively, trill calls have been proposed to indicate an increased degree of general arousal^49^ which in turn corroborates the anxious phenotype of SERT-KO rats.

Our results also demonstrated bidirectional changes in the proportion of other frequency modulated 50-kHz calls. Specifically, the percentage of multi-part calls (including mostly multi-step call category) was increased, whereas decreases were noted in the relative distribution of one-component calls (including mostly complex call category). While the exact meaning of this finding is unknown, it has recently been suggested that specific USVs subtypes may be associated with the particular type of behaviour^50^. For example, the increases in the rate of step calls have been linked to the onset of high-speed locomotion in pairs of rats tested in separated arenas^51^. In juvenile rats anticipating a play partner, multi-part call category was also most consistently associated with the high locomotor activity state, including running and jumping^50^. This vocalisation as being associated with anticipation of social contact appears not to be simply a “locomotion by-product” (widely discussed in^50,51^). We also cannot exclude the possibility that increased percentage of multi-component USVs in SERT-KO rats is related to increased partner following behaviour. In fact, positive correlation was found between the number of this call subtype and the duration of following the partner.

There were no differences between SERT-KO and SERT-WT rats in constant frequency 50-kHz calls of either short or long durations. It has been proposed that flat calls as being of non-affective nature may play distinct communicative roles than FM calls. As the emission of flat calls has been demonstrated during aggressive encounter^52^, the lack of differences in their emission found here is not surprising. The flat 50-kHz calls have also been interpreted as serving a role of contact calls with a social-coordinating function, as for example during social separation^53^. Interestingly, flats were negatively correlated with direct social contacts in wild-type control rats in the current study. This may suggest that this call category plays a role in spatial coordination of social behaviour also in our experimental set-up. Consequently, the absence of this correlation in SERT-KO rats may result from an atypical organization of social interactions.

In line with previous data^54^, the control rats emitted 50-kHz USVs when singly exploring the open field arena. Noteworthy, the number of emitted calls in control wild-type rats was positively correlated with the time spent in the center of the arena. As the decreased tendency to produce calls may be regarded as a measure of anxiety, the reduced number of 50-kHz USVs-emitting rats in the SERT-KO group as compared to wild-type controls may reflect a neophobic response associated with the anxious phenotype of those animals.

In conclusion, the present study demonstrates that genetic deletion of serotonin transporter not only leads to the deficits in social interaction and increased anxiety-like behaviour but also affects ultrasonic communication in rats. Further studies are needed to establish a more precise role for the 5-HT system in the emission of USVs.

## Materials and Methods

### Animals

The SERT knockout rat (Slc6a41Hubr) was generated by target-selected ENU-induced mutagenesis [for detailed description, see Smits *et al*.^55^] on a Wistar (Wistar ⁄ Crl) background]. Experimental animals were generated from incrosses between heterozygous SERT^+⁄–^ rats that have been outcrossed for at least ten generations. Animals were housed in a temperature-controlled (21 ± 1 °C) and humidity-controlled (40–50%) colony room under a 12/12 h light/dark cycle (lights on at 06:00 h). The rats were group-housed (4 rats/cage) with free access to food and water. Behavioural testing was performed during the light phase of the light/dark cycle. The experiments were conducted in accordance with the European Guidelines for animal welfare (2010/63/EU) and were approved by the II Local Ethics Committee for Animal Experiments at the Maj Institute of Pharmacology, Polish Academy of Sciences, Krakow, Poland.

### Experimental design

SERT-WT (SERT wild-type) and SERT-KO male rats (N = 33 and N = 27, respectively) at the age of 3 months were subjected to the open field test. The social interaction test was performed on the next day. After a two-week break, the rats’ social preference was assessed in a three-chamber test. A new cohort of rats (N = 24 and N = 25, for SERT-WT and SERT-KO, respectively) was used in the elevated plus maze test.

### Open field test

The experiments were conducted in an open field arena (length × width × height: 57 × 67 × 30 cm) made of black Plexiglas. The arena was dimly illuminated with an indirect light of 18 Lux. Rats were individually placed in the arena for 5 min. The distance travelled were automatically scored using the Any-maze® tracking system. Additionally, the time spent in the center of the open field was used as a measure of anxiety. The number of rearing episodes was manually scored by an experimenter.

### Social interaction test

The social interaction tests between male rats of the same genotype were conducted in the same open field arena as described above. The experiments were performed as previously reported^56^. The animals were handled and weighed, and the backsides of one half of the animals were marked with the Pentel permanent marker. The behaviour of the rats was recorded using a Sony light-amplification CCD camera placed above the arena and connected to a PC running Noldus MPEG recorder 2.1. An experimenter blinded to the treatment conditions analysed the videos off-line using Noldus Observer® XT, version 10.5.

On the test day, two unfamiliar rats of matched body weight (±5 g) were placed in the open field arena, and their behaviours were recorded for 10 min. Durations of the direct social contact and following behaviour were scored. The behaviour scored as a social contact included: sniffing (the rat sniffs the body of the conspecific), anogenital sniffing (the rat sniffs the anogenital region of the conspecific), social grooming (the rat licks and chews the fur of the conspecific) and climbing (the rat climbs over the back of the conspecific / stands on the back of the conspecific)). The following behaviour was scored when the rat moved toward and followed the other rat. The time of a social contact and following behaviour was measured for each rat separately. Because both animals in a pair yielded approximately equal scores, the time of social contact and following behaviour was expressed as a summed score for each pair of animals. The number of pairs used was N = 17 (SERT-WT) and N = 14 (SERT-KO). Because of an odd number of animals, one individual in each group was paired twice.

### USVs: recording and analysis

The rats’ vocalisations during the open field and social interaction tests were recorded as previously described by Potasiewicz *et al*.^57^ using an ultrasound microphone (CM16/CMPA, Avisoft Bioacoustics, Berlin, Germany) suspended 25 cm above the floor of the test area (tickling and SP test). The acoustic data were recorded using Version 1.5 Raven Pro Interactive Sound Analysis Software (The Cornell Lab of Ornithology Bioacoustics Research Program, Ithaca, NY, USA). The recordings of entire test sessions (5 min of the open field test and 10 min of the social interaction test) of all tested animals were analysed using Raven Pro software. Each call was manually marked on the computer screen and counted by an experienced user blinded to the treatment. Spectrograms were generated using a fast Fourier transform (FFT) length of 512 points and a time-window overlap of 75% (100% frame, Hamming window). Following parameters were assessed: a) the number of USVs, b) the peak frequency (the frequency with the highest amplitude measured in kHz), c) the bandwidth (difference between the highest and lowest frequencies; a measure of frequency modulation expressed in kHz), and d) the average duration of the call (length of the call measured in milliseconds)^58^.

Based on the acoustic call features, we also manually divided the calls into the following general types: short calls, flat calls with a near-constant frequency and frequency-modulated calls (^57^, detailed description in Table 1). The frequency modulated calls were subsequently classified as: the trills, one-component calls (predominantly complex calls and also ramp and inverted-U calls) and multi-component calls (predominantly multi-step calls and also step up, step down and composite calls)^4,7^. The detailed call classification was not conducted for the open field test due to the low number of emitted USVs and negligible representation of calls across selected categories.

**Table 1:** Classification of rat ultrasonic vocalisation subtypes.

50-kHz Subtype Name	Duration	Bandwidth	50-kHz Subtype Description
Short	Short, (<12 ms)	Typically narrow, <6-kHz	Short dot
Flat	Long (>12 ms)	Narrow, <6-kHz	Long line
Trill	Typically very long (>30 ms)	Typically very broad, >15-kHz	Rhythmic waves of ups and downs
Multi-component	Long (>12 ms)	Broad, >6-kHz	Calls that comprise two or more sounds
One-component	Long (>12 ms)	Highly variable, ~5 to 30 kHz	Variable changes with ascending/descending constant pattern

### Sociability and social novelty preference test

The procedure was adapted from Moy *et al*.^59^ and conducted with our modifications as previously described by Nikiforuk *et al*.^56^. The experiments were performed in a large open field arena (length x width x height: 100 × 60 × 30 cm) made of black Plexiglas that were divided into three compartments. Dividing walls were made from clear Plexiglas with arched openings (width × height: 10 × 12 cm) allowing access into each chamber. The apparatus was dimly illuminated with an indirect light of 18 Lux.

The testing started with a 10-min habituation to the apparatus 24 hours before the test. Rats were placed in the middle part of the apparatus and allowed to explore all three chambers. In the sociability phase, an unfamiliar rat (i.e., that had no previous contact with the tested rat) was enclosed in a cylindrical wire cage (height × diameter: 25 × 15 cm) that allowed nose contact between the bars but prevented fighting. The cage with the stimulus rat was placed in the middle of one of the outer compartments; the second outer compartment contained an empty wired cage. The tested rat was placed in the middle compartment of the apparatus and had free access to both outer compartments, i.e., the chamber with an empty wire cage and the chamber with a stimulus rat (a) for 5 min. Subsequently in the next phase, the rats were tested for another 5-minute session to quantitate social novelty preference. The test rat had a choice between the first, already investigated unfamiliar rat (rat a) and the novel unfamiliar rat (rat b).

The amount of time spent in each chamber was measured during sociability and social novelty preference test sessions. Rats that spend less than 5 s on exploration of one of the compartments were excluded from the analysis. The locations of both wire cages in the compartment on the left or right side of the apparatus were counterbalanced across the groups. Ten age-matched wild-type unfamiliar rats were acclimatised to the wire cage before the test, and they were alternatively used as a stimulus rat a or rat b within the experiments. The behaviour of the rats was recorded by a camera placed above the apparatus and connected to the Noldus MPEG. Videos were analysed manually off-line using the Noldus program The Observer® XT, version 10.5.

### Elevated plus-maze test

The procedure was adapted from Pellow and File^60^ and was conducted with our modifications as previously described by Nikiforuk *et al*.^61^.

The apparatus made of Plexiglas and elevated to the height of 50 cm, consisted of two open arms (40 × 12 cm) and two enclosed arms (40 × 12 × 20 cm) placed at 90^o^ to each other and extended from a central platform (12 × 12 cm). The experiments were conducted under low-intensity light (30 Lux). The test was initiated by placing a rat on the central platform of the maze facing an open arm. Testing lasted for 5 min and time spent in open and closed arms and the number of open and closed arm visits were recorded using the Any-maze® tracking system. The percentage of time spent in the open arms of the maze and the percentage of open arm visits served as measures of anxiety.

### Statistics

For consistency, all graphs represent median, 25th to 75th percentiles (boxes) and the full span of all data points (whiskers). Depending on the results of normality (Wilk-Shapiro test), data were analysed with either parametric or nonparametric tests. Differences between groups were analysed using Student’s t-test (when data were normally distributed) or Mann-Whitney U test (in case data were not normally distributed). Social interaction and sociability/social preference data were analysed using mixed design ANOVAs with genotype as a between-subject factor and behaviour type (or compartment) as a repeated measure followed by the Newman-Keul’s post-hoc tests. Chi^2^ test was used to test differences between proportions. All pairwise p values were corrected for multiple testing using the Benjamini-Hochberg procedure (Supplementary Table 1 S4)^62^. Spearman rank correlation test was used to analyse correlations between open field test and/or social behaviour measures, and the number of USV calls. All tests were two-tailed with the significance level set at p < 0.05. The statistical analyses were performed using Statistica 10.0 for Windows.

## Supplementary information

Supplementary Dataset 1-3.

Supplementary Info.

## Data Availability

All data generated during this study are included in this article and its supplementary file.
