# Supplementary material for: Serotonin transporter deficiency alters socioemotional ultrasonic communication in rats
Source: Sci Rep. 2019 Dec 30;9:20283. doi: 10.1038/s41598-019-56629-y (PMC6937290; doi:10.1038/s41598-019-56629-y)
Supplement: Supplementary file 1 — Supplementary Dataset 1-3. [file 41598_2019_56629_MOESM1_ESM.docx]

**Serotonin transporter deficiency alters socioemotional ultrasonic communication in rats.**

Joanna Golebiowska^1^, Małgorzata Hołuj^1^, Agnieszka Potasiewicz^1^, Diana Piotrowska^1^, Agata Kuziak^1^, Piotr Popik^1^, Judith R. Homberg^2^, Agnieszka Nikiforuk^1*^

**Supplementary materials:**

**Supplement 1**

**Supplement 2**

**Supplement 3**

**Supplement 1. 50-kHz USVs emission during the open field test.**

**Figure 1 S1. Distribution of vocalising and non-vocalising rats.**

Data are presented as a part of total number of animals in the group (N=33 and N=27 for SERT-WT and SERT-KO, respectively).

**Figure 2 S1. The total number of emitted 50-kHz calls in vocalizing rats.**

Data are presented as median (horizontal line), interquartile range (box) and minimum and maximum values (whiskers). N=24 (SERT-WT) and N=12 (SERT-KO) 50-kHz vocalising rats per group.

**Table 1 S1.** **Acoustic characteristic of 50-kHz calls.**

| Genotype | Duration  (ms) | Peak Frequency  (kHz) | Bandwidth  (kHz) | Emitting/  non-emitting |
| --- | --- | --- | --- | --- |
| SERT-WT | 26.53±2.46 | 60.69±1.02 | 8.64±1.04 | 24/9 |
| SERT-KO | 31.54±2.16* | 62.99±10.0 | 11.73±1.27* | 12/15* |
| Mann-Whitney  U Test | Z=2.097  P=0.036 | Z=1.459  NS | Z=2.064  P=0.039 | Chi2=4.95  P=0.026 |

Data are presented as the mean and S.E.M. Analysis was conducted only for rats emitting 50-kHz USVs (SERT-KO: N=24 and SERT-WT: N=12). Symbols: *p<0.05. The grayed rows indicate significant intergenotype differences.

**Supplement 2. USVs emission during the social interaction test.**

**Figure 1 S2. SERT-KO rats did not differ from controls in the number of ultrasonic calls.**

The total number of emitted calls. Data are presented as median (horizontal line), interquartile range (box) and minimum and maximum values (whiskers). 22-kHz calls are presented only for vocalising rats, see the result section.

**Table 1 S2. Acoustic characteristic of 50-kHz calls.**

| Call type | Genotype | Duration  (ms) | Peak Frequency  (kHz) | Bandwidth  (kHz) | Emitting/  non-emitting |
| --- | --- | --- | --- | --- | --- |
| SHORT | SERT-WT | 8.16±0.68 | 63.79±1.2 | 4.42±0.18 | 17/0 |
|  | SERT-KO | 7.98±0.10 | 66.77±0.97 | 4.81±0.21 | 14/0 |
| FLAT | SERT-WT | 32.76±8.73 | 53.58±2.76 | 2.10±0.11 | 13/4 |
|  | SERT-KO | 25.73±2.59 | 56.01±1.58 | 2.11±0.06 | 13/1 |
| TRILL | SERT-WT | 53.66±2.75 | 69.07±1.17 | 21.76±1.39 | 16/1 |
|  | SERT-KO | 49.14 ±1.49 | 68.43±0.58 | 18.29±0.73 | 14/0 |
| MULTI-PART | SERT-WT | 32.93±1.23 | 65.95±2.04 | 15.22±0.68 | 17/0 |
|  | SERT-KO | 32.68±1.40 | 68.97±2.64 | 15.20±0.77 | 14/0 |
| ONE-COMPONENT | SERT-WT | 28.35±2.44 | 58.75±0.91 | 7.48±0.27 | 17/0 |
|  | SERT-KO | 25.19±0.79 | 60.14±0.91 | 7.59±0.39 | 14/0 |

Data are presented as the mean and S.E.M. Analysis was conducted only for rats emitting specific call categories.

**Table 2 S2. Correlation between the duration of social behaviour and the number of 50-kHz calls.**

| **Call subtype [n]** | **SOCIAL CONTACT** | | **FOLLOWING** | |
| --- | --- | --- | --- | --- |
|  | **SERT-WT** | **SERT-KO** | **SERT-WT** | **SERT-KO** |
| **5O-kHz calls (total)** | -0.283 | 0.006 | 0.152 | 0.552* |
| **SHORT** | -0.261 | -0.002 | 0.103 | 0.723** |
| **FLAT** | -0.686** | -0.019 | 0.081 | 0.241 |
| **TRILLS** | -0.045 | 0.134 | 0.347 | 0.213 |
| **MULTI-PART** | -0.235 | 0.033 | 0.149 | 0.547* |
| **ONE-COMPONENT** | -0.407 | 0.011 | 0.164 | 0.508 |

Spearman’s r values are presented in the table. * p < 0.05. ** p < 0.01. The grayed rows indicate significant correlation.

**Supplement 3. Sociability and social novelty preference test.**

**Figure 1 S3. Number of entries into each compartment during the test for sociability (a) and preference for social novelty (b).**

Data are presented as median (horizontal line), interquartile range (box) and minimum and maximum values (whiskers).

Results:

There were no significant differences between SERT-WT and SERT-KO rats for number of entries into the two chambers during either the test for sociability (ANOVA interaction: F[1,58]=0.007; NS) or the test for novelty preference (ANOVA interaction: F[1,51]=0.883; NS).

**Figure 2 S3.** Total number of entries during the test for sociability (a) and preference for social novelty (b).

Data are presented as median (horizontal line), interquartile range (box) and minimum and maximum values (whiskers).

Results:

There were no significant differences between SERT-WT and SERT-KO rats for the total number of entries during either the test for sociability (t=1.92, df=58, p=0.059) or the test for novelty preference (t=2.01, df=51, p=0.051).

**Table S3. Correlation between the distance travelled in the open field test and the total number of entries during the sociability and social novelty preference tests.**

|  | sociability | novelty |
| --- | --- | --- |
| SERT-WT | 0.004 | 0.318 |
| SERT-KO | 0.039 | -0.298 |

Pearson’s r values are presented in the table
