# Supplementary material for: Serotonin transporter deficiency alters socioemotional ultrasonic communication in rats
Source: Sci Rep. 2019 Dec 30;9:20283. doi: 10.1038/s41598-019-56629-y (PMC6937290; doi:10.1038/s41598-019-56629-y)
Supplement: Supplementary file 2 — Supplementary Info. [file 41598_2019_56629_MOESM2_ESM.docx]

**Serotonin transporter deficiency alters socioemotional ultrasonic communication in rats.**

Joanna Golebiowska^1^, Małgorzata Hołuj^1^, Agnieszka Potasiewicz^1^, Diana Piotrowska^1^, Agata Kuziak^1^, Piotr Popik^1^, Judith R. Homberg^2^, Agnieszka Nikiforuk^1*^

**Supplementary materials:**

**Supplement 4**

**Table 1 S4.** The Benjamini-Hochberg multiple-testing correction.

| **RANK** | **TEST** | p value | correction |
| --- | --- | --- | --- |
| 1 | OF_distance | 0,00005400 | 0,000972 |
| 2 | 3-chamber sociability WT | 0,00011800 | 0,001062 |
| 3 | 3-chamber sociability KO | 0,00011800 | 0,000708 |
| 4 | OF_center | 0,00028500 | 0,001283 |
| 5 | USV type_one comp | 0,00032200 | 0,001159 |
| 6 | 3-chamber novelty WT | 0,00145500 | 0,004365 |
| 7 | USV type_multi part | 0,00180200 | 0,004634 |
| 8 | USV type_trills | 0,00274400 | 0,006174 |
| 9 | OF_rearings | 0,00490500 | 0,009810 |
| 10 | SI_following | 0,00802700 | 0,014449 |
| 11 | SI_contact | 0,00836600 | 0,013690 |
| 12 | USV_50_ OF | 0,02600000 | 0,039000 |
| 13 | USV_22_ OF | 0,09900000 | 0,137077 |
| 14 | USV_22 kHz | 0,14956000 | 0,192291 |
| 15 | USV_50 kHz | 0,16515100 | 0,198181 |
| 16 | USV type_short | 0,39342400 | 0,442602 |
| 17 | USV type_flat | 0,39342400 | 0,416567 |
| 18 | 3-chamber novelty KO | 0,43922000 | 0,439220 |

P values were corrected for multiple testing using the Benjamini–Hochberg method.
